# German nationwide inpatient data on the post-COVID-19 syndrome: an update

**DOI:** 10.3389/fpubh.2026.1774115

**Published:** 2026-02-17

**Authors:** Nike Walter, Markus Rupp, Thilo Hinterberger, Thomas H. Loew, Ines Rittmann

**Affiliations:** 1 Department for Psychosomatic Medicine, University Hospital Regensburg, Regensburg, Germany; 2 Department of Trauma, Hand, and Reconstructive Surgery, University Hospital Giessen, Giessen, Germany

**Keywords:** epidemiology, healthcare costs, inpatient care, post-COVID syndrome, treatment procedures

## Abstract

**Background:**

Post-COVID syndrome (PCS) continues to pose a serious public health issue, with persistent symptoms requiring ongoing medical care after the acute phase of infection. Despite its clinical significance, data on its burden on hospital systems and healthcare costs have been limited. This study updates the national analysis of PCS-related hospitalizations in Germany for the year 2023, focusing on patient demographics, primary diagnoses, procedures, and inpatient costs.

**Methods:**

Using nationwide data from the Institute for the Hospital Remuneration System (InEK) and the German Diagnosis-Related Groups (G-DRG), the study identified patients with PCS via the diagnosis code “U09.9!” as a secondary diagnosis. Parameters included age, sex, diagnoses, procedures, length of stay (LOS), and direct cost estimates. Incidence rates were calculated using official population data.

**Results:**

There were 17,209 PCS-related hospital admissions, translating to 48.9 cases per 100,000 people. Women represented 60% of cases, with peak prevalence in the 55–60 age group. Common diagnoses were chronic fatigue, dyspnea, and general malaise; procedures mainly involved respiratory and neurological evaluations. Only 4% required care dependency assessments. The average LOS was 8.3 days (SD 11.6 days). LOS showed a right-skewed distribution. The median LOS was 7.1 days [interquartile range (IQR): 3.9–7.9 days]. Total inpatient costs reached €67.9 million. Compared to early-pandemic data, the number of PCS-related hospitalizations decreased substantially in 2023, while diagnostic complexity and inpatient resource utilization remained high.

**Conclusion:**

PCS continues to challenge healthcare systems, underlining the need for ongoing research, policy adjustments, and resource planning.

## Introduction

The COVID-19 pandemic has had a profound and enduring impact on global health, with both its acute and long-term effects shaping healthcare priorities worldwide. Among these, the persistence of symptoms following the resolution of acute SARS-CoV-2 infection, commonly referred to as Post-COVID Syndrome (PCS) has emerged as a major public health concern. PCS is characterized by a heterogeneous constellation of symptoms, ranging from persistent fatigue, pain, dyspnea, and cognitive dysfunction to more severe manifestations, including cardiovascular, neurological, and pulmonary complications (1–3). Since the emergence of PCS as a recognized condition (4), substantial efforts have been made to quantify its prevalence, characterize its symptoms, and understand its clinical trajectory and treatment approaches (5, 6).

At least 65 million individuals globally have been estimated to be affected by long COVID, based on an assumption that 10% of the more than 651 million documented COVID-19 cases worldwide have developed the condition (7). A pooled global prevalence of 0.43 was reported by a recent meta-analysis (8). In 2021, we published a comprehensive analysis of PCS based on nationwide German inpatient data, highlighting the condition’s prevalence, symptomatology, and economic impact during the early phases of the pandemic (9).

This study updates nationwide inpatient PCS data using 2023 records. Our objectives are to first reassess the prevalence and demographic distribution of PCS, second identify associated comorbidities, treatment modalities and third evaluate its ongoing economic healthcare burden. This updated analysis seeks to inform stakeholders, guiding healthcare policies, resource allocation, and the development of targeted management strategies.

## Methods

This cross-sectional study utilized hospital data obtained from the Institute for the Hospital Remuneration System (InEK GmbH), which oversees Germany’s performance-based hospital reimbursement system. This system, established under Section 17b of the German Hospital Financing Act (KHG), is based on the German Diagnosis-Related Groups (G-DRG) system, where inpatient cases are assigned a fixed reimbursement amount depending on their diagnosis and treatment.

Data were accessed via the InEK Data Browser (available at: https://datenbrowser.inek.org/login), focusing on annual ICD-10 diagnosis codes for the year 2023. Specifically, the number of patients diagnosed with “U09.9! post-COVID-19 condition” as a secondary diagnosis was identified. Each record represents a single inpatient hospital admission. Due to the aggregated and anonymized nature of the InEK dataset, repeated hospitalizations of the same individual could not be identified and therefore cannot be excluded.

For the analysis, several parameters were extracted. Demographic data included the number of cases stratified by age group and sex. The most frequently documented primary diagnoses associated with post-COVID-19 condition were identified using ICD-10-GM codes. Incidence rates were calculated based on population data provided by the Federal Statistical Office of Germany (Destatis) (https://www-genesis.destatis.de/, Accessed Nov 24, 2024). Here, the number of inhabitants in each of the 16 German federal states was considered by year of birth. To assess medical procedures, commonly performed Operation and Procedures Keys (OPS)-coded interventions such as diagnostic tests, imaging, and therapeutic treatments were analyzed. Additionally, length of stay (LOS) data were examined. A cost analysis was performed calculating mean reimbursement per case based on distribution across different DRG categories. The cost analysis was conducted from the perspective of the statutory hospital reimbursement system. Reported costs represent DRG-based reimbursement amounts and reflect direct inpatient hospital costs, including diagnostic procedures, therapeutic interventions, and hospital stay, but excluding outpatient care, rehabilitation, and indirect costs. All costs are reported in euros (€) and are based on the official German Diagnosis-Related Groups (G-DRG) tariff catalogue for the year 2023. In the German DRG system, inpatient cases are reimbursed using a flat-rate payment model, whereby each hospital admission is assigned to a specific DRG based on the primary diagnosis, secondary diagnoses, procedures performed, patient age, and length of stay. Secondary diagnoses, such as post-COVID-19 condition (ICD-10-GM code U09.9!), may influence case severity classification, complexity levels, and reimbursement amounts, particularly when associated with increased diagnostic or therapeutic resource utilization. Median values and dispersion measures were derived from DRG-level mean values weighted by the number of cases per DRG, as patient-level length-of-stay and reimbursement distributions are not available in the InEK Data Browser. Accordingly, the reported reimbursement amounts should be interpreted as standardized proxies for inpatient resource use rather than as exact hospital-level or societal costs.

### Results

**Figure 1:**
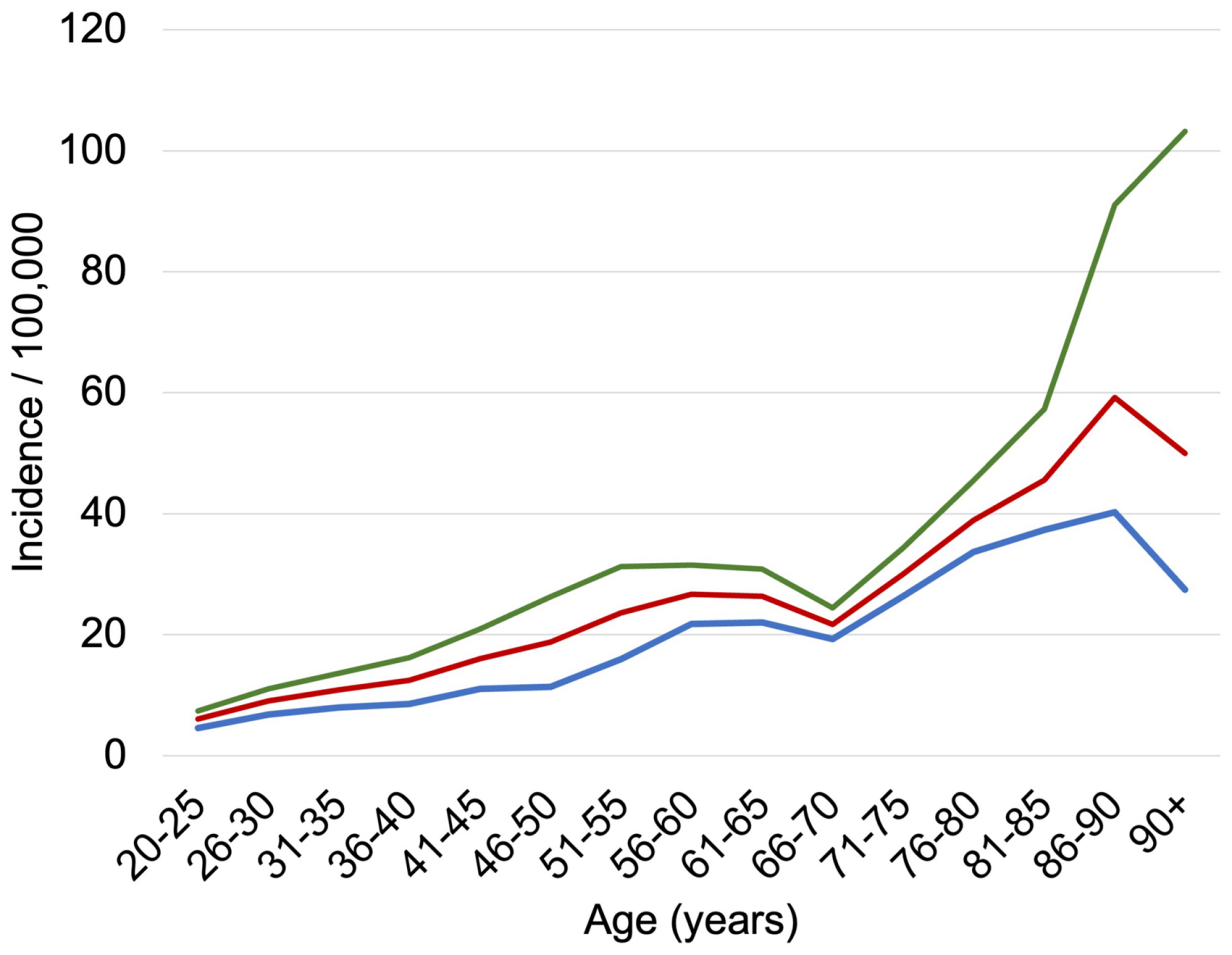
Incidence of PCS inpatient cases per 100,000 inhabitants, standardized by age and sex. The blue line represents male patients, the red line represents female patients, and both genders are depicted in green.

**Figure 2:**
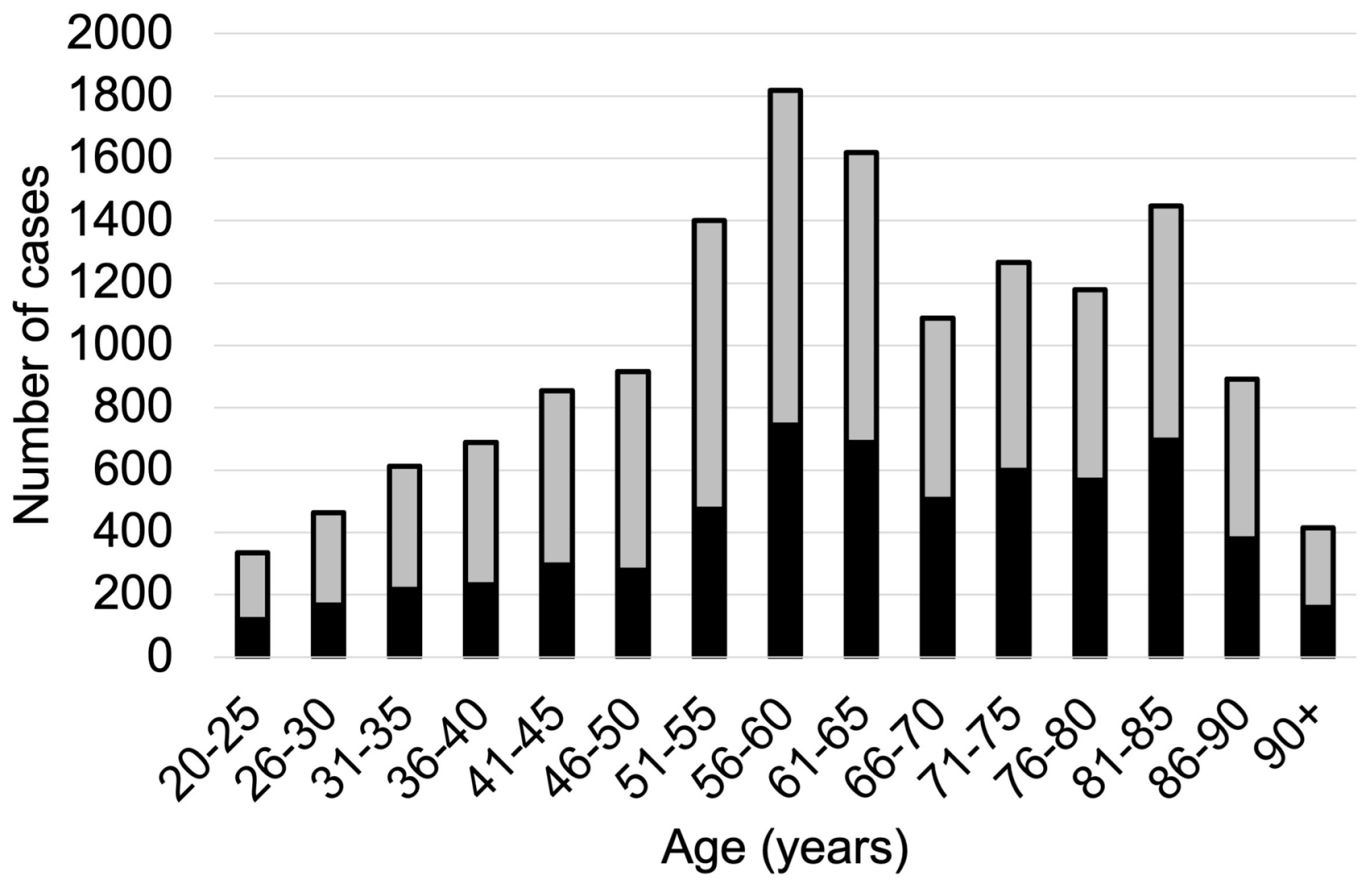
Number of cases in 5-year increments. Male patients are illustrated in black, female cases are shown in grey.

In 2023, 17,209 inpatient cases were recorded with a PCS diagnosis, corresponding to an incidence of 48.9 per 100,000 inhabitants (Figure 1). Among them, 6,856 (40%) were male and 10,353 (60%) were female, with the highest prevalence observed in the 55–60 age group (Figure 2). The mean length of stay (LOS) for hospitalized PCS patients was 8.3 days, with a standard deviation of 11.6 days. LOS showed a right-skewed distribution. The median LOS was 7.1 days [interquartile range (IQR): 3.9–7.9 days].

A total of 1,945 distinct primary diagnoses were associated with PCS. The most frequently documented conditions included chronic fatigue syndrome, dyspnea, malaise, and fatigue, alongside other common diagnoses such as fibromyalgia, chronic pain disorders, pneumonia, and dizziness (Table 1).

**Table 1:** Twenty most frequent primary diagnoses in PCS patients.

Code	Primary diagnosis	Cases	Percentage
G93.3	Chronic fatigue syndrome	2,277	13.23%
R06.0	Dyspnea	814	4.73%
R53	Malaise and fatigue	783	4.55%
R26.8	Other and unspecified gait and mobility disorders	319	1.85%
F45.41	Chronic pain disorder with somatic and psychological factors	286	1.66%
M79.70	Fibromyalgia: multiple localizations	211	1.23%
F48.0	Neurasthenia	188	1.09%
J12.8	Pneumonia due to other viruses	174	1.01%
E86	Volume depletion	157	0.91%
G62.80	Critical-illness polyneuropathy	141	0.82%
J84.10	Other interstitial lung diseases with fibrosis (without acute exacerbation)	133	0.77%
R42	Dizziness and giddiness	129	0.75%
J96.00	Acute respiratory insufficiency, unspecified elsewhere: type I (hypoxic)	123	0.71%
R51	Headache	123	0.71%
J18.9	Pneumonia, unspecified	117	0.68%
I50.01	Secondary right heart failure	115	0.67%
F06.7	Mild cognitive disorder	112	0.65%
I50.13	Left heart failure: symptoms with mild exertion	110	0.64%
G47.31	Obstructive sleep apnea syndrome	108	0.63%
I26.9	Pulmonary embolism without acute cor pulmonale	107	0.62%

The most commonly performed procedures involved respiratory diagnostics, such as whole-body plethysmography, thoracic CT with contrast, and computer-assisted 3D image analysis. Additional frequent procedures included CO diffusion capacity testing, cranial imaging, and care dependency assessments. Neurological evaluations, including electroencephalography (EEG) and neurography, were also frequently documented, alongside therapeutic interventions such as geriatric rehabilitation and tracheobronchoscopy (Table 2). This indicates that while PCS frequently leads to hospitalization, severe functional dependency affects a smaller subset of patients.

**Table 2:** Twenty most commonly performed medical procedures in PCS patients.

Code	Procedure	Cases	Percentage
1–710	Whole-body plethysmography	2,543	14.78%
3–222	Thoracic CT with contrast	1851	10.76%
3–990	Computer-assisted image data analysis with 3D evaluation	1761	10.23%
1–711	Determination of CO diffusion capacity	1,587	9.22%
9–984.7	Need for care: care level 2	1,508	8.76%
3–200	Native cranial CT	1,276	7.41%
8–930	Monitoring of respiration, heart, and circulation without pulmonary arterial or central venous pressure measurement	1,270	7.38%
1–204.2	Examination of the cerebrospinal fluid system: lumbar puncture for CSF extraction	1,189	6.91%
9–984.8	Need for care: care level 3	1,189	6.91%
8–975.23	Naturopathic complex treatment: 14–20 treatment days and <2,520 treatment min. or 10–13 days and ≥1,680 min.	1,117	6.49%
1–632.0	Diagnostic esophagogastroduodenoscopy: normal findings	1,027	5.97%
3–800	Native MRI of the skull	1,020	5.93%
3–202	Native thoracic CT	983	5.71%
1–207.0	Electroencephalography (EEG): routine EEG (10–20 system)	961	5.58%
1–206	Neurography	897	5.21%
8–550.1	Geriatric early rehabilitative complex treatment: ≥14 treatment days and ≥20 therapy sessions	893	5.19%
3–225	Abdominal CT with contrast	812	4.72%
9-984.b	Need for care: application for care level classification	736	4.28%
3–820	MRI of the skull with contrast	705	4.10%
1–208.2	Registration of evoked potentials: somatosensory (SSEP)	661	3.84%
1–620.01	Diagnostic tracheobronchoscopy: flexible instrument with bronchoalveolar lavage	609	3.54%
1–620.00	Diagnostic tracheobronchoscopy: flexible instrument without further measures	543	3.16%
9–984.6	Need for care: care level 1	531	3.09%
1–843	Diagnostic aspiration from the bronchus	521	3.03%
3–035	Complex differential diagnostic ultrasound of the vascular system with quantitative evaluation	475	2.76%

All distributional measures were derived from DRG-level mean values weighted by the number of cases per DRG, as patient-level distributions are not available in the InEK Data Browser. The most frequently coded G-DRG was Z65Z (Complaints, Symptoms, Other Anomalies, and Follow-up Treatment), accounting for 17.7% of cases (3,039 patients). The mean reimbursement per inpatient case was €3,947.97, with substantial variability across DRG categories (standard deviation: €916.28), reflecting heterogeneity in clinical complexity and resource utilization. Extrapolating these costs to the 17,209 recorded PCS patients, the total direct healthcare costs for PCS treatment were estimated at €67,940,616.

## Discussion

This cross-sectional study provides an update for the year 2023 on (i) the demographics of hospitalized PCS patients, (ii) the most frequently documented primary diagnoses associated with PCS, (iii) the most commonly performed medical procedures, and (iv) the annual direct healthcare costs related to PCS hospitalizations. A key strength of this analysis is its foundation on nationwide healthcare data from Germany, one of the largest countries in the European Union, ensuring a comprehensive and representative assessment. Compared to earlier phases of the pandemic, the absolute number of hospitalized PCS cases declined (9), suggesting evolving disease dynamics and healthcare utilization patterns.

### Hospitalization and demographics

With over 17,000 recorded hospitalizations, PCS remains a clinically relevant condition in Germany. The demographic distribution of hospitalized PCS patients reveals a notable gender imbalance, with women comprising 60% of cases. This aligns with international findings suggesting higher susceptibility to long-term post-viral sequelae among females (10, 11). Other studies conducted in different countries report around 53% to be female (12, 13). Possible explanations to the greater extent of females developing PCS can be found in the literature pointing towards sex-specific innate and adaptive immune correlates (14, 15). The age group most affected was between 55 and 60 years, consistent with prior data identifying middle-aged and older adults as high-risk populations for prolonged post-infectious sequelae. Notably, compared to prior data published, the number of hospitalized cases decreased from 29,808 by 43% (9).

### Comorbidities and symptom spectrum

The clinical presentation of PCS was highly diverse, with a variety of primary diagnoses documented. The most frequent included chronic fatigue syndrome, dyspnea, malaise, fibromyalgia, chronic pain, and neurocognitive symptoms—indicating multi-system involvement. These are consistent with existing literature on PCS symptomatology (16–18).

Importantly, these findings reflect not only residual effects of acute COVID-19, but also secondary complications such as autonomic dysfunction, post-exertional malaise, and psychological distress. This constellation of symptoms often co-occurs with pre-existing conditions or is exacerbated by them, highlighting the need for a multidisciplinary treatment approach that integrates pulmonology, neurology, internal medicine, pain management, and psychosomatic care. Rehabilitation strategies and coordinated outpatient follow-up are likely essential to address the persistent functional impairments in this patient population (19, 20).

### Diagnostic procedures and clinical management

The wide range of medical procedures underscores the substantial diagnostic burden PCS imposes on hospitals. This diversity in clinical presentation illustrates the diagnostic complexity of PCS and reinforces the need for comprehensive and systematic clinical evaluation (21, 22).

Respiratory diagnostics such as whole-body plethysmography and CO diffusion testing were frequently used. Extensive application of thoracic CT, MRI, EEG, and lumbar puncture further reflects the multi-organ complexity of PCS. These interventions suggest that clinicians routinely pursue broad differential diagnoses to assess unexplained respiratory, neurological, and systemic complaints. Given the lack of definitive biomarkers or confirmatory tests, PCS remains a diagnosis of exclusion (23, 24). Thus, future studies should aim to identify specific diagnostic markers or imaging signatures that can support early recognition, stratification, and tailored management of PCS.

Notably, care dependency assessments were documented in a considerable proportion of cases, suggesting that PCS can correlate with a significant loss of function and autonomy in a subset of patients. The need for formal evaluation of care requirements reflects the real-world burden not only on patients but also on families, caregivers, and social support systems—a factor that warrants consideration in future healthcare planning and resource allocation (25).

### Healthcare costs

With nearly €67.9 million direct inpatient costs PCS represents a notable financial strain on the healthcare system. These figures varied widely depending on case complexity and resource intensity. In addition to direct medical costs, PCS is associated with substantial indirect economic consequences, including work absenteeism, reduced productivity, and prolonged disability. Previous estimates place the indirect financial burden in Germany at over €3.4 billion (26). These findings underscore the urgent need for healthcare policymakers, insurers, and public health stakeholders to develop coordinated response strategies. Sustainable financing models, investment in multidisciplinary care infrastructure, and support for return-to-work programs will be essential to mitigate both the clinical and economic impact of PCS. The transferability of the reported cost estimates to other healthcare systems should be interpreted with caution. The German DRG-based reimbursement system is characterized by standardized flat-rate payments that reflect average resource use rather than actual expenditure at the individual hospital level. As a result, cost estimates may differ in countries with alternative financing models, such as fee-for-service systems, global hospital budgets, or mixed public–private reimbursement structures. Nevertheless, the observed magnitude of inpatient resource utilization highlights that PCS imposes a substantial economic burden across healthcare systems, irrespective of the specific reimbursement framework. While absolute cost figures may not be directly comparable across countries, the relative intensity of diagnostic procedures, prolonged hospital stays in a subset of patients, and sustained inpatient utilization suggest that PCS represents a relevant cost driver in diverse health system contexts. Healthcare systems relying on fee-for-service reimbursement or global hospital budgets may experience different absolute cost estimates; however, the observed intensity of inpatient diagnostics and prolonged hospitalization in a subset of patients suggests comparable resource implications across systems.

### Health system implications

The sustained number of PCS-related hospitalizations and the broad range of diagnostic procedures underscore that PCS represents a structural challenge for hospital systems rather than a transient post-pandemic phenomenon. The frequent use of respiratory and neurological diagnostics, combined with prolonged lengths of stay in a subset of patients, suggests that current inpatient care pathways may not be optimally adapted to the needs of PCS patients. Strengthening outpatient specialized PCS clinics, interdisciplinary rehabilitation programs, and standardized diagnostic pathways may reduce unnecessary hospital admissions and improve continuity of care.

### Limitations

This study has several limitations that should be considered when interpreting the findings. First, the analysis is based on secondary administrative data from the InEK database, which is primarily designed for hospital reimbursement rather than epidemiological research. Since hospital billing data are primarily intended for financial and operational purposes, clinical accuracy may be affected by variations in coding practices. The accuracy of PCS diagnoses depends on proper documentation and ICD-10 coding by hospital staff, introducing a potential risk of misclassification, underreporting, or overcoding. Additionally, PCS remains a relatively new diagnosis, and its clinical definition and diagnostic criteria are still evolving, which may have led to heterogeneous coding across different hospitals. Financial and administrative incentives within DRG-based reimbursement systems thus, may influence diagnostic coding practices, potentially affecting PCS case identification.

Second, this study only includes inpatient cases, meaning that individuals who were diagnosed and treated in outpatient settings were not captured. Since many patients with PCS are managed in primary care, specialized outpatient clinics, or through rehabilitation programs, this exclusion leads to selection bias, overrepresenting more severe cases requiring hospitalization. Consequently, the true burden of PCS in the general population may be underestimated, as milder cases and those managed without hospitalization are not included in the dataset. It is worth noting that in Germany, 248,104 patients received outpatient treatment in the fourth quarter of 2023, according to data from the Zentralinstitut für die kassenärztliche Versorgung (Zi). These figures are based on nationwide pseudonymized billing data from statutory health insurance physicians, compiled across health insurance providers in accordance with § 295 of the German Social Code Book V (SGB V) (27). Third, patient-level characteristics and clinical details such as symptom severity, laboratory values, or imaging findings are not included in the dataset. The analysis does not account for individual risk factors such as pre-existing comorbidities, lifestyle factors, vaccination status, or prior COVID-19 severity, which could significantly influence hospitalization risk and outcomes. Additionally, socioeconomic variables, such as employment status, income, or access to specialized care, are not captured, limiting the ability to assess potential disparities in healthcare access and treatment patterns.

Further, while this study specifically aimed to estimate direct inpatient costs using G-DRG reimbursement data, it does not capture indirect costs or outpatient care expenses. As a result, the economic burden of PCS is likely underestimated, since costs associated with primary care visits, rehabilitation, mental health services, long-term disability, and productivity loss are not included.

## Conclusion

In conclusion, this study provides comprehensive insights into the inpatient burden of PCS in Germany, demonstrating that PCS remains a significant public health challenge with substantial healthcare costs and resource utilization. PCS hospitalizations predominantly affected middle-aged individuals, with a higher prevalence among women, and were frequently associated with chronic fatigue syndrome, dyspnea, neurological symptoms, and functional impairments. The frequent use of respiratory and neurological diagnostics underscores the multi-system nature of PCS, while the proportion of care dependency assessments highlights the long-term disability risk faced by many patients. From an economic perspective, the study confirms PCS as a costly condition, with total direct inpatient costs nearing €68 million. Taken together, these findings emphasize the need for long-term healthcare planning, standardized care pathways, and sustained research efforts to address the persistent clinical and economic burden of PCS.

## Data Availability

Publicly available datasets were analyzed in this study. This data can be found at: the datasets generated and analyzed during the current study are publicly available via the InEK Data Browser at https://datenbrowser.inek.org/. Additional aggregated data supporting the findings of this study are available from the corresponding author on reasonable request.
